# Saharan dust and association between particulate matter and case-specific mortality: a case-crossover analysis in Madrid (Spain)

**DOI:** 10.1186/1476-069X-11-11

**Published:** 2012-03-08

**Authors:** Julio Díaz, Aurelio Tobías, Cristina Linares

**Affiliations:** 1 National School of Public Health, Carlos III Institute of Health. Avda.Monforte de Lemos, 5. 28029 Madrid, Spain; 2 Institute of Environmental Assessment and Water Research (IDAEA), Spanish Council for Scientific Research (CSIC). C/JordiGirona, 18-26. 08034 Barcelona, Spain; 3 Department of Environmental Epidemiology and Cancer, National Centre of Epidemiology, Instituto de Salud Carlos III, Madrid, Spain; 4 CIBER Epidemiología y Salud Pública (CIBERESP), Madrid, Spain

## Abstract

**Background:**

Saharan dust intrusions are a common phenomenon in the Madrid atmosphere, leading induce exceedances of the 50 μg/m^3^- EU 24 h standard for PM_10_.

**Methods:**

We investigated the effects of exposure to PM_10 _between January 2003 and December 2005 in Madrid (Spain) on daily case-specific mortality; changes of effects between Saharan and non-Saharan dust days were assessed using a time-stratified case-crossover design.

**Results:**

Saharan dust affected 20% of days in the city of Madrid. Mean concentration of PM_10 _was higher during dust days (47.7 μg/m^3^) than non-dust days (31.4 μg/m^3^). The rise of mortality per 10 μg/m^3 ^PM_10 _concentration were always largely for Saharan dust-days. When stratifying by season risks of PM_10_, at lag 1, during Saharan dust days were stronger for respiratory causes during cold season (IR% = 3.34% (95% CI: 0.36, 6.41) versus 2.87% (95% CI: 1.30, 4.47)) while for circulatory causes effects were stronger during warm season (IR% = 4.19% (95% CI: 1.34, 7.13) versus 2.65% (95% CI: 0.12, 5.23)). No effects were found for cerebrovascular causes.

**Conclusions:**

We found evidence of strongest effects of particulate matter during Saharan dust days, providing a suggestion of effect modification, even though interaction terms were not statistically significant. Further investigation is needed to understand the mechanism by which Saharan dust increases mortality.

## Background

The increased levels of particulate matter in a given geographical area are mainly influenced by the intrusion of natural origin such as those related to the advection of dust from the desert [1]. Obviously, the areas which are closer to the great deserts are the most affected by this type of events. In the case of the Saharan desert, Southern European countries are largely influenced by dust intrusions [2]. Dust particles can also be transported over long distances by atmospheric circulation, reaching the Near East end the Americas [3].

Saharan dust events can contribute to exceedances of PM_10 _daily European Union Limit of 50 μg/m^3 ^[2,4]. Moreover, these particles can carry biological material that makes them potentially harmful to health [5]. Among the diseases that would be affected by dust intrusions are those related to exacerbations of respiratory diseases, especially paediatric asthma [6-8].

Recently, this subject is rising from studies conducted in Southern Europe, although they have not given consistent results. In Spain, a study conducted in Barcelona [9] reported strongest effects of coarse particles (PM_10-2.5_) on total daily mortality during Saharan dust intrusions. In Italy, a study conducted in Rome [10] also found strongest effects of PM_10 _and coarse particles on daily mortality for cardiovascular causes, although others in Emilia-Romagna region [11] did not found an increase of risk of total and case-specific mortality due to PM_10 _during Saharan dust days. Lastly, studies in Athens did not found effect modification of PM_10 _on total and case-specific mortality [12], although they did found an increase of risk on paediatric asthma exacerbation [8].

The city of Madrid has high levels of pollution of anthropogenic origin, which are strongly increased those days with Saharan dust intrusions, and its geographical and meteorological conditions that did not facilitate the dispersion of pollutants. The aim of this study is to evaluate the effect of Saharan dust on the association between particulate matter and daily case-specific mortality in Madrid.

## Methods

### Setting

The city of Madrid constitutes a dense metropolitan area located on the central region of Spain. The main emission source of atmospheric pollutants is road traffic. Its geographical setting has climatological conditions directly linked to emission levels, with frequent anticyclonic situations in summer and winter, which impede the dispersion of pollutants. Saharan dust events on the central region of Spain generally occur 50 to 100 days per year, mainly between spring and autumn, mostly caused by an atmospheric depression West or Southwest of Portugal or by anticyclonic conditions over Algeria inducing Southern winds over the Iberian Peninsula [1].

### Data

Daily mortality was obtained from the Madrid Regional Inland Revenue Department (*Consejería de Hacienda*), which is the department responsible for mortality registry, from 1st January 2003 to 31st December 2005. The outcomes of interest were organic causes except accidents (ICD-10: A00-R99), respiratory causes (ICD-10: J00-J99), circulatory causes (ICD-10: I00-I99) and cerebrovascular mortality (ICD-10: I60-I69).

Daily mean concentrations of PM_10 _were obtained from the automated network of the Madrid's City Comprehensive Air-Pollution Monitoring, Forecasting and Information System (*Red de Control de Contaminación Atmosférica del Ayuntamiento de Madrid*). Daily mean levels of gaseous pollutants (NO_2_, SO_2_) and O_3 _were also collected.

Saharan dust intrusions, a dichotomous indicator variable used to show whether or not there had been a Saharan dust intrusion in the Madrid atmosphere on the date in question, with the pertinent data being obtained from the Directorate-General for Environmental Quality & Assessment (*Dirección General de Calidad and Evaluación Ambiental*) at the Ministry for the Environment and Rural & Marine Habitats (*Ministerio de Medio Ambiente y Medio Rural y Marino*) (available at: http://www.calima.ws/, last accessed February 17th, 2012). Briefly, an integrated methodological approach was used to identify days on which air masses from the Sahara-Sahel region were transported to the central region of Spain through back-trajectory analysis (Hysplit model), information from NRL SKIRON and BSCDREAM dust maps, and satellite images provided by the NASA SeaWiFS [1].

Daily mean temperature and relative humidity registered at the Madrid-Retiro Observatory, situated at city centre, were recorded by the National Meteorology Agency (*Agencia Nacional de Meteorología*).

### Design and statistical analysis

The association of daily concentrations of PM_10 _with daily mortality was investigated using a case-crossover design [13]. This uses the day on which the outcome of interest (mortality) occurs as a case day. Exposure on case days is compared with exposure on days on which the outcome of interest does not occur (control days) [14]. A time-stratified approach used to represent exposure on control days from the same day of the week, month, and year as case days, minimising bias from time trends in the exposure series and from other short-term time-varying confounders [14].

We fitted a basic Poisson regression model to the daily mortality data that included potential confounders: weather, influenza, epidemics and time trends and seasonality. A natural cubic spline with 3 degrees of freedom was built to adjust for the potential confounding of mean temperature (4-day average) and a linear term to adjust for relative humidity (4-day average). We also controlled for gaseous pollutants and O_3_, by means of a 4-day average linear term, and for public holidays and influenza epidemics by means of categorical variables. Finally, we included a three-way interaction term between day of the week, month, and year to control for both seasonality and time trends. This choice was motivated by the need to replicate the adjustment made by the case-crossover design with the time-stratified approach for the selection of control days [15]. To take into account possible overdispersion of daily counts of deaths, we used quasi-likelihood estimation.

The effects of exposure to PM_10 _were examined for the same day (lag 0) to 4 days after exposure (lag 4). Estimated effects are reported as IR% and 95% CI associated with a 10 μg/m^3 ^increase of PM_10_. A dummy variable was created for the presence or absence of Saharan dust outbreaks at exposure days and its interaction with PM_10 _was fitted to test for effect modification by Saharan dust outbreaks. Because of most of Saharan dust intrusions occurs in Spain during the hot season, as previously reported [9], separate estimates were undertaken for the period May-September (warm season) and October-April (cold season). All analyses were done using Stata, release 11, statistical software (StataCorp, College Station, TX, 2010).

## Results

During 2003-2005, Saharan dust affected 20% of days in the city of Madrid (218 days). The proportion of days with a dust episode was maximal in the hot season (28.4%) and lowest in the cold one (11.3%). The mean concentration of particles was higher during dust days than during non-dust days (47.7 μg/m^3 ^versus 31.4 μg/m^3^), while for gaseous pollutants did not differ substantially whereas levels of O_3 _were slightly larger during dust days. As expected, mean temperature was also higher during dust days (20.4°C versus 13.4°C), even though relative humidity was higher during non-dust days (46.2% versus 56.4%). Case specific mortality was similarly distributed between Saharan dust and non-dust days. Overall, organic causes represent 48% of the total mortality, circulatory causes 30%, respiratory causes 15% and cerebrovascular causes 7% (Table 1).

**Table 1:** Summary statistics for daily case-specific mortality, PM_10 _and weather variables during Saharan dust and non-dust days in Madrid, 2003-2005.

	Non-Saharan dust days (n = 878)							Saharan dust days (n = 218)						
	**Mean**	**(sd)**	**Min**.	**P_25_**	**P_50_**	**P_75_**	**Max**.	**Mean**	**(sd)**	**Min.**	**P_25_**	**P_50_**	**P_75_**	**Max**.

Daily deaths														

Organic	61.5	(12.2)	32	53	60	75	109	59.6	(10.0)	34	53	59	66	88

Respiratory	9.6	(4.5)	0	7	9	12	32	8.8	(3.7)	2	6	8	11	22

Circulatory	18.8	(5.5)	5	15	18	22	40	17.9	(5.0)	6	14	17	22	32

Cerebrovascular	4.3	(2.1)	3	4	6	12	34	4.4	(2.1)	0	3	4	6	10

Particulate matter														

PM_10 _(μg/m^3^)	31.4	(15.4)	8	19	29	40	117	47.7	(19.1)	11	35	46	56	150

Gaseous pollutants														

NO_2 _(μg/m^3^)	59.7	(18.6)	19	47	57	72	133	60.7	(14.0)	28	51	60	72	100

SO_2 _(μg/m^3^)	12.2	(5.8)	5	8	10	16	36	10.1	(3.3)	5	8	9	12	25

Ozone (μg/m^3^)	33.2	(17.8)	5	18	33	48	79	41.4	(17.2)	6	29	42	55	89

Weather														

Temperature(°C)	13.4	(7.7)	-2.2	6.8	12.2	20.3	30.1	20.4	(6.8)	2.4	14.0	22.1	25.9	30.2

Humidity(%)	56.4	(20.0)	13	39	57	74	96	46.2	(19.9)	15	28	43	63	94

**Figure 1:**
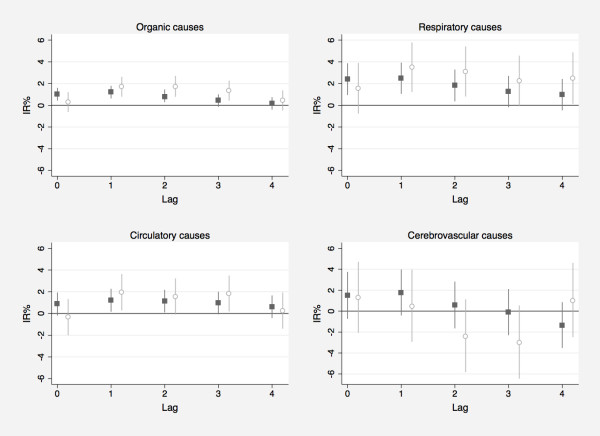
**Percentage increases of case-specific mortality for an increase of 10 μ/m^3 ^of PM_10_, from lag 0 to lag 4, during Saharan dust (with circle) and non-dust days (back squares) with 95% confidence interval (vertical lines)**.

The rise of mortality per 10 μg/m^3 ^PM_10 _concentration increase was stronger, and statistically significant (p < 0.05) at lag 1 for both, Saharan dust and non-dust days (Figure 1), unless for cerebrovascular causes. Risks were always larger for Saharan dust-days providing a suggestion of effect modification, even though interaction terms between Saharan dust and non-dust were not statistically significant (Table 2).

**Table 2:** Association between particulate matter and daily case-specific mortality during Saharan dust and non-dust days, by season*

	Non-Saharan dust days		Saharan dust days		
					p-value for interaction
(Lag 1)	%IR	(95% CI)	%IR	(95% CI)	
Organic					

Cold	**1.32**	**(0.67, 1.97)**	0.26	(-1.00, 1.54)	**0.150**

Warm	0.76	(-0.61, 2.15)	**2.53**	**(1.01, 4.08)**	**0.066**

Whole year	**1.21**	**(0.63, 1.79)**	**1.70**	**(0.79, 2.62)**	0.372

Respiratory					

Cold	**2.87**	**(1.30, 4.47)**	**3.34**	**(0.36, 6.41)**	0.792

Warm	-0.59	(-4.22, 3.17)	-0.05	(-3.95, 4.01)	0.830

Whole year	**2.50**	**(1.05, 3.93)**	**3.48**	**(1.22, 5.79)**	0.462

Circulatory					

Cold	0.83	(-0.33, 2.00)	-0.16	(-2.41, 2.14)	0.453

Warm	**2.65**	**(0.12, 5.23)**	**4.19**	**(1.34, 7.13)**	0.391

Whole year	1.21	(0.16, 2.27)	1.95	(0.29, 3.64)	0.408

Cerebrovascular					

Cold	2.14	(-0.32, 4.70)	-2.78	(-7.43, 2.10)	0.079

Warm	0.90	(-4.06, 6.12)	3.35	(-2.34, 9.37)	0.500

Whole year	1.76	(-0.42, 3.98)	0.46	(-2.93, 3.96)	0.528

*Cold season: October to April, warm season: May to September.

Table 2 shows results also stratified by season at strongest lag previously found (lag 1). Risk of PM_10 _for organic causes was larger, and statistically significant, during cold season for non-dust days (1.32% [95% CI: 0.67, 1.97]) whilst during warm season risk was larger for Saharan dust days (2.53% [95% CI: 1.01, 4.08]) also providing evidence of effect modification with interaction terms marginally significant (p = 0.150 and p = 0.066 for cold and warm season, respectively). Moreover, during cold season effects of PM_10 _were stronger, and statistically significant, for respiratory causes being the larger risk for Saharan dust days (3.34% [95% CI: 0.36, 6.41] versus 2.87% [95% CI: 1.30, 4.47]) while for warm season effects were stronger for circulatory causes and risk was larger for Saharan dust days (4.19% [95% CI: 1.34, 7.13] versus 2.65% [95% CI: 0.12, 5.23]). However, none of the interaction terms were statistically significant. No effects were found for cerebrovascular causes for both seasons.

## Discussion

Saharan dust transport events reaching the city of Madrid showed strongest effects of daily concentrations of PM_10 _on case-specific mortality due to organic, respiratory and circulatory causes. Therefore, during Saharan dust-days these effects were larger in the cold season for respiratory causes and in the warm season for circulatory ones.

The distribution of daily concentrations of PM_10 _reported in our city, with higher levels on Saharan dust intrusion days, are consistent with the increased contribution of particulate matter from the Sahara because of the natural advections of this. Saharan dust intrusions tend to occur more frequently in the warmer months, which agrees with the frequency of synoptic-scale weather situations [16], mainly characterized by higher temperatures and low concentrations in relative humidity. Similar synoptic behaviour and frequency of Saharan dust intrusions have been described in the study conducted in the Emilia-Romagna region [11], while in Athens are less frequent and have no clear seasonal pattern [12].

The effects of PM_10 _on case specific mortality found in our study agreed with those previously reported in other European cities. The APHEA-2 study reported that an increase in PM_10 _by 10 μg/m^3 ^was associated with increases of 0.76% in cardiovascular deaths and 0.58% in respiratory deaths [17]. However, only few studies have assessed the effect of Saharan dust events on the association between PM_10 _and daily mortality, reporting inconsistent findings. Our results, showing an increase in mortality due to organic, respiratory and circulatory causes during Saharan dust days, are similar to those recently found in Rome [10]. However, another study conducted in the Emilia-Romagna region [11] concluded that Saharan dust outbreaks are an independent risk factor that increases respiratory mortality. On the contrary, a latest study in Athens [12] did not found effect modification of PM_10 _on total and case-specific mortality due to dust events. In addition, studies focused on daily mortality related to dust of non-Saharan dust origin, like those in Washington [18] and Seoul [19], also fail to find any effect on Saharan dust events.

Currently, some studies have been focus on the effect of coarse-fraction (PM_10-2.5_). A study conducted in Barcelona [9] found a stronger effect of PM_10-2.5 _on daily total mortality during Saharan dust days, as well as the previously cited in Rome [10] that also found evidence of stronger effects on cardiovascular mortality. However, the health effects of coarse particles are still controversial, since most of the available studies were not able to isolate an independent effect of coarse particles from fine particles [20]. Furthermore, previous studies of African dust size distributions showed that long distant transport reduces the amount of heavier and larger particles in the Saharan air masses, thus increasing the relative contribution of smaller particles [21]. These data suggested than the usual definition of coarse fraction (PM_10-2.5_) usually adopted in air pollution monitoring also in epidemiological studies should be use with caution as specific marker of Saharan dust transports, which are probably better overall described by PM_10-1 _[11]. However, we did not consider other PM fractions since from a previous study we did not found differences between Saharan and non-Saharan dust days for PM_2.5 _[22], and no PM_1 _levels were monitored during the study period. Nevertheless, the issue of harmful effects of Saharan dust has recently been addressed by taking PM_10 _into account, as recently did in the Emilia-Romagna region [11] and in Athens [12]. Thus, our study is entirely comparable with those in terms of outcome and exposure evaluated, increasing the ongoing evidence on the mortality induced by the influx of Saharan dust in southern Europe.

A major strength of our study is that seasonal dependency, only conducted previously in the study conducted in the Emilia-Romagna region [11], has also been addressed. We found strongest effects of PM_10 _in daily mortality by circulatory causes during the warm season, and on respiratory causes during cold season. This confirms the seasonal pattern of case-specific mortality in Madrid, described in earlier studies more than two decades ago [23]. This seasonal dependency was not found in the Emilia-Romagna region [11]. This could indicate that the activity of biological agents in Saharan air masses differs depending on the season and the environmental conditions experienced by air masses travelling from the Sahara to Southern Europe [4,11]. An interaction with other environmental factors not included in the analyses could be another feasible explanation.

As limitation, we have not addressed for contextual socioeconomic status and other potential modifiers, such as age and sex. However, the study design that would have meant a spatial adjustment had been very different to that conducted in our study [24]. From a pathophysiological point of view there it is well known that the elderly group is most vulnerable to the short-term effects of air pollution on health [25], we have not considered age groups due to it was not available. Furthermore, as well as in previous studies [10-12], we do not have available data on chemical and biological components or on the copresence of other pollutants in particulate matter during Saharan dust days, so cannot determine specific components that could account for stronger estimated effects on Saharan dust days, or confirm the natural or anthropogenic origin of particulate matter. Only the study in Barcelona [9] reported data on chemical composition of fine and coarse particles for Saharan dust and non-dust days, concluding that although coarse particles seem to be more hazardous during Saharan dust days, differences in chemical composition did not explain these observations. Inconsistencies in the existing evidence suggests that characteristics associated with the short-term effects of desert dust should be further studied, with special attention to their composition, biological properties, and anthropogenic components that may be associated with the dust particles [10].

However, the results found in our study jointly with the knowledge of a possible biological mechanism that indicates that particulate material from the desert carry biological material which makes them particularly harmful to health [5,26,27], as well as the findings from other Mediterranean cities, such as Barcelona [9] and Rome [10], with similar settings like Madrid, make obvious the need to conduct further studies with a similar methodology in Southern Europe.

## Conclusions

Our study adds to existing evidence of short-term effects of PM_10 _on the risk of daily case-specific mortality, especially for organic, respiratory and circulatory causes. During Saharan dust intrusion we also observed effects of PM_10 _on mortality due to respiratory causes in the cold season and to circulatory causes in the warm one. These shows the need that air quality standards must consider the potential harmful effects of dust from natural sources. Furthermore, additional research is needed to understand the mechanism by which particulate matter from desert sources can increase mortality.

## Abbreviations

PM_10: _Particulate matter < 10 μm in aerodynamic diameter; PM_10-2.5: _Particulate matter size between 10 and 2.5 μm in aerodynamic diameter; PM_10-1: _Particulate matter size between 10 and 1 μm in aerodynamic diameter; NO_2: _Nitrogen dioxide; SO2: Sulphur dioxide; O_3: _Ozone; ICD-10. International Classification of Diseases 10th revision; IR%: Percentage increases in risk of death; CI: Confidence interval; p: P-value.

## Competing interests

The authors declare that they have no competing interests.

## Authors' contributions

JD, AT and CL designed the original study, AT analyzed data, and JD, AT and CL interpreted the data, and wrote the manuscript. All authors read and approved the final manuscript.
